# Structured crowdsourcing enables convolutional segmentation of histology images

**DOI:** 10.1093/bioinformatics/btz083

**Published:** 2019-02-06

**Authors:** Mohamed Amgad, Habiba Elfandy, Hagar Hussein, Lamees A Atteya, Mai A T Elsebaie, Lamia S Abo Elnasr, Rokia A Sakr, Hazem S E Salem, Ahmed F Ismail, Anas M Saad, Joumana Ahmed, Maha A T Elsebaie, Mustafijur Rahman, Inas A Ruhban, Nada M Elgazar, Yahya Alagha, Mohamed H Osman, Ahmed M Alhusseiny, Mariam M Khalaf, Abo-Alela F Younes, Ali Abdulkarim, Duaa M Younes, Ahmed M Gadallah, Ahmad M Elkashash, Salma Y Fala, Basma M Zaki, Jonathan Beezley, Deepak R Chittajallu, David Manthey, David A Gutman, Lee A D Cooper

**Affiliations:** 1 Department of Biomedical Informatics, Emory University School of Medicine, Atlanta, GA, USA; 2 Department of Pathology, National Cancer Institute, Cairo, Egypt; 3 Department of Medicine, Cairo University, Cairo, Egypt; 4 Egyptian Ministry of Health, Cairo, Egypt; 5 Department of Medicine, Ain Shams University, Cairo, Egypt; 6 Department of Medicine, Menoufia University, Menoufia, Egypt; 7 Department of Pathology, Medical Research Institute, Alexandria University, Alexandria, Egypt; 8 Department of Medicine, Chittagong University, Chittagong, Bangladesh; 9 Department of Medicine, Damascus University, Damascus, Syria; 10 Department of Medicine, Mansoura University, Mansoura, Egypt; 11 Department of Medicine, Zagazig University, Zagazig, Egypt; 12 Department of Medicine, Batterjee Medical College, Jeddah, Saudi Arabia; 13 Department of Medicine, Suez Canal University, Ismailia, Egypt; 14 Kitware Inc., Clifton Park, NY, USA; 15 Department of Neurology, Emory University School of Medicine, Atlanta, GA, USA; 16 Department of Biomedical Engineering, Emory University, Atlanta, GA, USA

## Abstract

**Motivation:**

While deep-learning algorithms have demonstrated outstanding performance in semantic image segmentation tasks, large annotation datasets are needed to create accurate models. Annotation of histology images is challenging due to the effort and experience required to carefully delineate tissue structures, and difficulties related to sharing and markup of whole-slide images.

**Results:**

We recruited 25 participants, ranging in experience from senior pathologists to medical students, to delineate tissue regions in 151 breast cancer slides using the Digital Slide Archive. Inter-participant discordance was systematically evaluated, revealing low discordance for tumor and stroma, and higher discordance for more subjectively defined or rare tissue classes. Feedback provided by senior participants enabled the generation and curation of 20 000+ annotated tissue regions. Fully convolutional networks trained using these annotations were highly accurate (mean AUC=0.945), and the scale of annotation data provided notable improvements in image classification accuracy.

**Availability and Implementation:**

Dataset is freely available at: https://goo.gl/cNM4EL.

**Supplementary information:**

Supplementary data are available at *Bioinformatics* online.

## 1 Introduction

Accurate segmentation of tissue regions in histology images is a challenging problem with important applications in computational pathology. The ability to accurately delineate tissue regions can provide important information for computational diagnosis, prognostication, assessments of treatment response and investigations of cancer biology. The problem of *semantic segmentation*, or exhaustive pixel-level classification of tissues, is particularly challenging. While deep-learning methods have demonstrated promising results in general semantic image segmentation problems, these encoder-decoder convolutional architectures require large training datasets to generalize well. Generating annotated histology datasets with adequate scale presents significant challenges, especially when careful delineation of regions or structures is required, and the lack of annotated histology remains a significant barrier in the growth of computational pathology. Semantic segmentation is particularly challenging, as complete labeling of the scene is required. Generating a meaningful number of annotations requires engaging with multiple experts, and even experienced pathologists will exhibit some inter-rater discordance. Annotations need to be captured on many images, as remarkable histologic variations can be observed even within a single lab, and variations in tissue processing (fixation, staining, mounting) and imaging have a strong influence on image texture and color. Data augmentation techniques are often used when training networks to simulate this variation by artificially manipulating the color and contrast of images with some success, reducing the annotation burden. Still, a pathologist with significant clinical demands often cannot produce enough annotations on their own to adequately train deep-learning models for challenging applications like semantic segmentation. Interfaces for viewing and annotating whole-slide histology images, collaborative review and data management are also a critical element in engaging pathologists to scale the production of accurate ground truth.

Crowdsourcing has been extensively used in general non-medical tasks, and has been shown to markedly speed and scale the process of image annotation (Su, 2012). In the life sciences, crowd-based approaches based on gamification or micropayments enabled successful scaling of biological annotations (Hughes *et al.*, 2018). In pathology, however, the value of crowdsourcing is not immediately apparent due to the complexity and subjectivity of tasks, scale of whole-slide scans (necessitating custom large-scale viewers and annotation platforms), and domain expertise needed; a recent systematic review found that almost all crowdsourcing articles in the pathology literature focused on malaria diagnosis and relatively simple scoring of immunohistochemical biomarkers (Alialy *et al.*, 2018). Recent work has established the value of crowdsourcing for nucleus detection and segmentation microtasks in hematoxylin and eosin stained images, and that research fellows and some non-pathologists (NPs) are able to reach acceptable concordance with senior pathologists (SPs) (Irshad *et al.*, 2015, 2017). This work was based on a limited number of slides (10), focused on small regions of interest (400×400 up to 800×800 pixels), and did not explore more challenging tasks such as semantic segmentation. Moreover, this work did not investigate how to organize participants and leverage their various experience levels, and how technology can facilitate feedback and collaboration between more and less experienced participants to improve crowdsourcing efficiency and accuracy.

To address some of these issues, we investigate the use of crowdsourcing in the context of semantic segmentation of breast cancer images. This task is widely regarded as the most laborious and challenging type of ground truth generation (Kovashka *et al.*, 2016). We describe our experience using web-based technology to facilitate an international crowdsourcing effort, and in expanding this effort to include junior pathology residents (JPs) and medical students. We also describe how training and directed feedback using web-based tools like the Digital Slide Archive (DSA) can be instrumental in streamlining the annotation and review process. Our annotation efforts focus on triple-negative breast cancer (TNBC), an aggressive genomic subtype that comprises ∼15% of breast cancer cases (Plasilova *et al.*, 2016).

## 2 Materials and methods

### 2.1 Dataset description

The dataset used in this study consists of 151 hematoxylin and eosin stained whole-slide images (WSIs) corresponding to 151 histologically-confirmed breast cancer cases. These images of formalin-fixed paraffin-embedded tissues were acquired from the Cancer Genome Atlas, with triple-negative status determined from clinical data files. A representative region of interest (ROI) was selected within each slide by the study coordinator, a medical doctor, and approved by a SP. The mean ROI size was 1.18 mm^2^ (SD = 0.80 mm^2^). ROIs were selected to be representative of predominant region classes and textures within each slide. Very large ROIs were avoided to prevent degradation in quality due to participant fatigue (Irshad *et al.*, 2015). Regions with high tumor density were selected whenever possible, for two reasons: (i) to maximize the proportion of ROI occupied by tumor; (ii) to minimize the need to distinguish normal/inflammatory cells and cancerous tissue, an exhaustive process requiring expertise not expected from NPs.

### 2.2 Participant recruitment and training

**Fig. 1.**
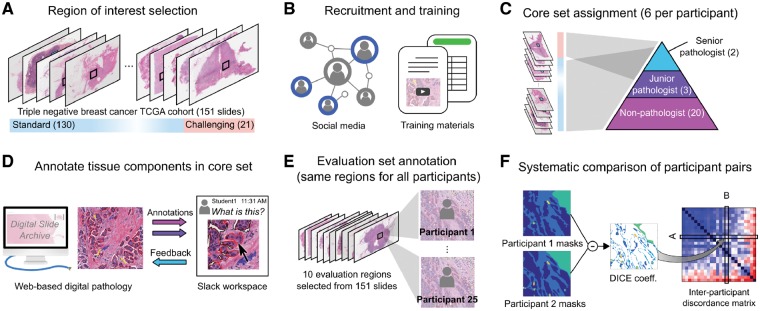
Study overview. (**A**) Slides from the TNBC cohort were reviewed for difficulty and the study coordinator selected a single representative ROI in each slide. (**B**) Participants were recruited on social media from medical student interest groups. Documentation and instructional videos were developed to train participants in breast cancer pathology and the use of DSA annotation tools. A spreadsheet lists slide-level descriptions of histologic features for each of the 151 images to aid in training. (**C**) Participants were each assigned six slides based on experience. Challenging slides were assigned to faculty/pathology residents, while standard slides were distributed among all participants. (**D**) The DSA was used by participants to draw the outlines of tissue regions in their assigned slides/ROIs. A Slack workspace enabled less experienced users to ask questions and receive guidance from the more experienced users. (**E**) Ten evaluation ROIs were identified in the slides and were annotated by all participants in an unsupervised manner to enable inter-participant comparisons. (**F**) Agreement between each pair of participants was evaluated using the Dice coefficient to generate an inter-participant discordance matrix

The study workflow is illustrated in Figure 1. Research interest groups on social media (including Facebook and LinkedIn) were used to recruit participants, who were asked to submit a resume and brief motivation statement to the study coordinator. A total of 25 participants, including 20 medical students, 3 JPs and 2 SPs were selected during recruitment. Throughout this manuscript, we use the following notation to denote the various participant classes: SP (senior resident or faculty); JP; NP. JPs were defined as pathology residents who have not finished their second year of residency training. Participants underwent a training session, composed of introductory videos and a detailed document describing guidelines, histological patterns to annotate, common pitfalls as well as instructions for using the DSA interface. Supplementary Table S1 illustrates sample instructions provided to participants to help improve and standardize the annotation process. Slack, an online team communication tool, was used for NPs to ask questions and to receive feedback from other participants and pathologists. Extensive feedback was provided on the first slide annotated by a participant, serving as a *de-facto* practical component of their training.

### 2.3 Structured crowdsourcing

We use the term *structured crowdsourcing* to refer to systematic assignment of tasks based on participant experience and expertise. SPs assisted in mentoring and correcting annotations made by NPs. Two types of ROIs were annotated: (i) a ‘core’ set comprising 151 large ROIs to be used for training and validating algorithms (ALs) and (ii) an ‘evaluation’ set comprising 10 smaller ROIs used to evaluate inter-participant concordance.

Each participant was asked to annotate 5–6 ROIs from the core set (uniquely assigned to the participant) and all 10 evaluation ROIs. ROIs from challenging slides (21 total) were assigned to SPs and JPs, whereas all other ROIs (130 total) were evenly distributed among the participants. Slides were considered challenging if a considerable fraction of the ROI was occupied by uncommon features like extensive tumor cell vacuolation, stromal epithelialization or stromal hyalinization. Throughout the study, SPs provided feedback and made corrections to the core slides annotated by the participants. Feedback was not provided on evaluation set ROIs to avoid biasing the analysis of inter-participant concordance.

### 2.4 The DSA annotation interface

**Fig. 2.**
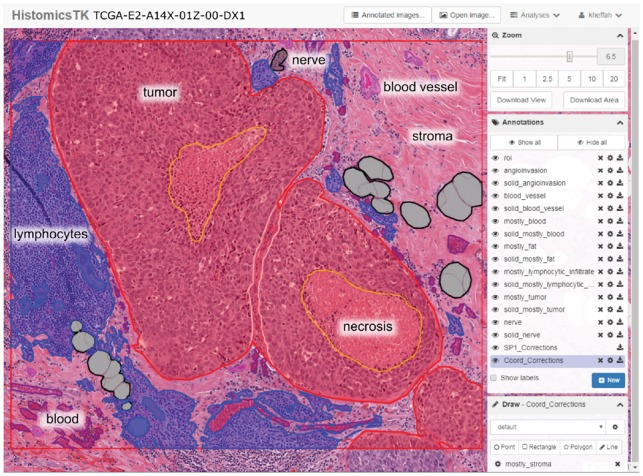
Screenshot of the DSA and HistomicsTK web interface. The main viewport allows panning and zooming within the slide. Annotations are grouped by class into layers (middle right panel) whose style properties like color and fill can be adjusted (bottom right panel). Other features include: controlling annotation transparency, an interactive mode to highlight individual annotations, and ability to download the WSI, regions of interest or annotations. Annotation properties can also be programmatically manipulated using the DSA API

The DSA is an open-source web-based digital pathology platform for the management, visualization and annotation of WSI datasets (Gutman *et al.*, 2013, 2017). A Docker software container along with instructions for creating a DSA instance is available at: https://github.com/DigitalSlideArchive/digitalslidearchive.info. Figure 2 shows a screenshot of the DSA interface used for annotation. This interface organizes annotations by region class (e.g. tumor, necrosis). Each class has a style that defines the rendering properties for its annotations including class names and boundary colors. These styles were pre-defined in a template by the study coordinator in consultation with an SP, and serve to improve the consistency of annotations across participants and to facilitate review. The DSA also provides a REST API for programmatic management of slide and annotation data that was used throughout the study to enable management of users, slide assignments, annotations, review and inter-participant concordance analyses.

### 2.5 Annotation review process

The following regions were annotated during crowdsourcing: (i) predominant classes including tumor, stroma, lymphocyte-rich regions and necrosis. (ii) Non-predominant classes including artifacts, adipose tissue, blood vessels, blood (intravascular or extravasated red blood cells), glandular secretions and extracellular mucoid material and (iii) challenging classes including plasma cells, mixed inflammatory infiltrates (e.g. neutrophils), normal ducts or acini, metaplastic changes (osteoid matrix, cartilaginous metaplasia, etc.), lymph vessels, skin adnexa, angioinvasion and nerves. Since stroma is the most prevalent component in many slides, it was considered to be the ‘default’ class and defined by absence of annotations. JPs and NPs were directed to focus their effort on annotating predominant classes and to ask for feedback on Slack when annotating non-predominant or challenging classes. Providing feedbacks publicly on the Slack channel allowed all participants to access and learn from each other’s questions and responses. The study coordinator and SPs reviewed all annotations for mistakes using two mechanisms: (i) providing feedback to the participants on the Slack channel and (ii) generating new correction overlay annotations that are patched on top of the original annotations (Supplementary Fig. S1). Two phases of review and corrections were used (Supplementary Methods and Supplementary Fig. S3).

### 2.6 Measuring annotation discordance

The polygonal coordinates are queried using the DSA server REST API and are converted to a mask image format offline, where pixel values encode region class (Supplementary Fig S1). The Inter-participant discordance was assessed for the 300 unique pairs of participants using their annotations on the evaluation set images. Discordance was measured using the Dice coefficient:

(1) $$\Delta_{i,j}=1-2\cdot \frac{\sum_{c=1}^{N_{c}}\left|I_{c}\cap J_{c}\right|}{\sum_{c=1}^{N_{c}}\left|I_{c}\right|+\left|J_{c}\right|}$$

where *i* and *j* are two participants, with corresponding masks *I*, *J*, composed of *c* binary channels, where $N_{c}\;$is the number of classes being considered. $\Delta_{i,j}\;$lies in the range [0, 1], where 0 indicates no discordance. Our analysis makes comparisons on the effect of experience level and feedback on annotation quality. We used two techniques to visualize discordance between participants. The first is a bi-clustered heatmap of the inter-participant discordance matrix that groups participants based on discordance profiles. The second is a multidimensional scaling (MDS) analysis of the discordance matrix that depicts participants as points in two-dimensional space and where proximity indicates concordance.

### 2.7 Semantic segmentation and classification models

A pre-trained fully convolutional VGG-16, FCN-8 network was trained to segment histology images into five classes: tumor, stroma, inflammatory infiltrates, necrosis and other classes (Long *et al.*, 2015). Shift and crop data augmentation was used to improve model robustness—see Supplementary Methods for details. Focusing on the 125 ROIs from infiltrating ductal carcinomas [the majority of TNBCs (Plasilova *et al.*, 2016)], we first applied color normalization to the RGB images of the ROIs (Reinhard *et al.*, 2001). Several different types of models were trained to evaluate different aspects of crowdsourcing:

Firstly, to investigate the effects of using crowdsourced versus single-expert annotations for training, we trained ‘comparison’ models for semantic segmentation. These models used annotations from evaluation set ROIs for training, and were evaluated on the post-correction core-set annotations (see Supplementary Fig. S2C).

Second, to evaluate peak accuracy, we trained ‘full’ models for semantic segmentation using the largest amounts of crowdsourced annotations possible. The full models were trained using annotations from core-set ROIs, assigning the ROIs from 82 slides (from 11 institutes) to the training set, and the ROIs from 43 slides (from seven institutes) to the testing set. Strict separation of ROIs by institute into either training or testing provides a better measure of how models developed with our data will generalize to slides from new institutions and multi-institute studies.

Finally, to evaluate the effect of training set size on the accuracy of predictive models, we developed ‘scale-dependent’ image classification models using varying amounts of our crowdsourced annotation data (Supplementary Fig S5). Since training hundreds of semantic segmentation models is time prohibitive, we instead trained classification models based on the pre-trained VGG-16 network to classify 224×224 pixel patches from the three predominant classes: tumor, stroma and inflammatory infiltration, using the same train/test assignment used in the semantic segmentation model (see details in Supplementary Methods).

## 3 Results

Our study produced a total of 50 057 polygonal annotations, including 3988 corrections. Following integration of the corrections (Supplementary Fig. S1B), a total of 20 340 polygonal annotations were extracted from the final mask images. The number of annotations within each ROI ranged from 11 to 541. Supplementary Table S2 describes the number of annotations by class, with the predominant classes representing more than 71% of the annotations. This data can be visualized in a public instance of the DSA at https://goo.gl/cNM4EL. Mask images derived from this data are used in training and validation are available at: *goo.gl/UoUm9w*. Further details can be found in the Supplementary Materials.

### 3.1 Annotation concordance is class-dependent

**Fig. 3.**
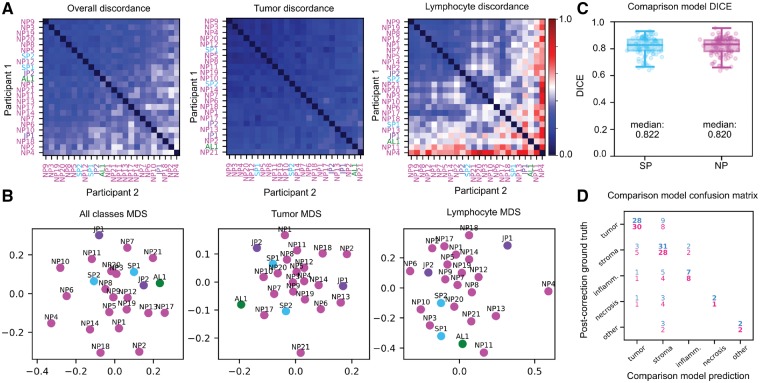
Evaluation slide set concordance and model accuracy. (**A**) Inter-participant discordance matrices for SP, JP, NP and AL. (**B**) 2-D MDS plots of participant discordance. (**C, D**) Testing accuracy and confusion of comparison models trained on evaluation set ROIs from SPs (cyan) and NPs (magenta), measured against post-correction masks from the core set. Confusion matrix values are percentages relative to total pixel count. (Color version of this figure is available at *Bioinformatics online*.)

Discordance varies significantly by class, and reflects the difficulty and subjectivity inherent in the classes (Fig. 3 and Supplementary Fig. S2). Tumor annotations were the least discordant, with 0.13 (SP–SP), 0.16 (NP–NP) and 0.15 (SP–NP). These results indicate that both the bias (SP–NP) and variance (NP–NP) of annotations made by NPs are lower when only the predominant class is considered (Mann–Whitney *P* = 3.66*e*−30 and *P* = 1.99*e*−168, for SP–NP and NP–NP, respectively).

SPs had low median discordance for tumor (0.13), stroma (0.19) and necrosis (0.09), and had relatively higher discordance for lymphocytic infiltration (0.48). The median discordance between NPs and SPs was 0.14, 0.27, 0.54 and 1.0 for tumor, stroma, lymphocyte infiltration and necrosis/debris, respectively. Similarly, the median discordance among NPs was 0.14, 0.33, 0.50 and 1.0 for tumor, stroma, lymphocytic infiltration and necrosis/debris, respectively. The high discordance for necrosis/debris reflects the fact that many participants either missed this class when it was truly present or misclassified stroma as necrosis.

Supplementary Figure S4A shows the pixel-wise average SP–NP discordance between NPs and pathologists for two typical regions. Most of the discordance for tumor occurs around the region boundary. On the other hand, discordance for lymphocytic infiltration and, to a lesser extent, necrosis/debris follows a more diffuse pattern, and is not limited to the region boundary.

### 3.2 Feedback improves annotation quality

There was some clustering of participants by experience level, with three of the more experienced participants (two SPs and one JP) being highly mutually concordant as seen in the MDS plot (Fig. 3). The median SP–SP discordance was 0.24, compared to 0.30 for NP–NP. Discordance for SP–NP comparisons lies in the middle of this range at 0.27. Predictions of a semantic segmentation AL trained on corrected annotations from independent institutions results in discordance values similar to those of SPs (Fig. 3); the median SP-AL discordance is 0.22 overall and 0.15 for tumor. Three primary mistakes observed during the correction of core-set annotations were: (i) imprecise region boundaries, (ii) region misclassification and (iii) missing annotations for non-predominant classes. Examples of common mistakes are presented in Supplementary Figure S4B. Discordance analysis results of the pre- and post-correction core-set annotations were consistent with trends observed in the evaluation set, but were notably lower. The median discordance between pre- and post-correction masks is 0.08 for all classes (SD = 0.30). This was significantly lower (Wilcoxon *P* = 8.77*e*−14) for binary tumor classification (0.01, SD = 0.11). Predominant classes had relatively low discordance, non-predominant classes had higher discordance, while challenging classes were almost always missing in NP annotations and were added by SPs in corrections.

The following are pre- and post-correction discordance values for other region classes (median ± SD): stroma (0.09 ± 0.15), lymphocytic infiltrate (0.08 ± 0.31), necrosis (0.25 ± 0.42), blood (0.02 ± 0.32), exclude (0.01 ± 0.42), fat (3.98*e*−4 ± 0.29), extracellular mucoid material (0.50 ± 0.50), glandular secretions (0.87 ± 0.42) and blood vessel (1.00 ± 0.28).

### 3.3 Accuracy of semantic segmentation models

The comparison semantic segmentation models had similar accuracy whether they were trained with NP annotations or SP annotations (median DICE = 0.820 versus 0.822, Fig. 3C and D). This result is consistent with the concordance results presented in Section 3.1.

**Fig. 4.**
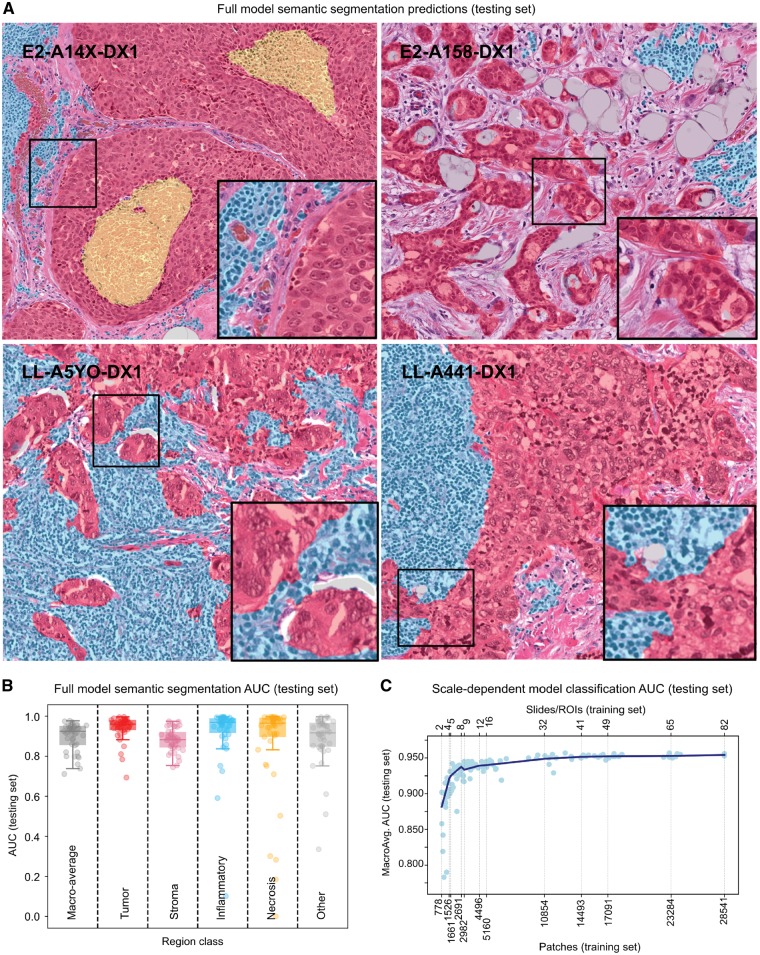
Model performance over the testing set. (**A**) Visualization of full semantic segmentation model predictions on testing set regions of interest. Color codes used: red (tumor); transparent (stroma); cyan (inflammatory infiltrates); yellow (necrosis). (**B**) Area under ROC curve for semantic segmentation algorithm, broken down by region class. (**C**) Effect of training sample size on scale-dependent patch classification models. Each point represents the macro-average AUC of a single model, trained on different sets of randomly selected slides. (Color version of this figure is available at *Bioinformatics online*.)

The segmentations generated by the full semantic segmentation model were highly accurate and concordant with human annotations of ROIs in the testing set (see Table 1 and Supplementary Fig. S6). The model predictions correspond well to region boundaries and are often more granular than human annotations (see Figure 4 and Supplementary Figs S7–S9). When misclassifications occur, they are generally due to the composition of the training set.

**Table 1.** Testing accuracy of full semantic segmentation model

	Mean AUC (SD)	DICE	Accuracy
Overall	0.945 (0.042) (micro)	0.888	0.799
Tumor	0.941 (0.058)	0.851	0.804
Stroma	0.881 (0.056)	0.800	0.824
Inflammatory	0.917 (0.150)	0.712	0.743
Necrosis	0.864 (0.237)	0.723	0.872
Other	0.885 (0.129)	0.666	0.670

Errors were found in uncommon or mixed patterns including: dense pure plasma cell infiltrates (classified as tumor), acellular hyaline stroma (classified as tumor) and necrotic regions containing dense inflammatory infiltrates (classified as infiltrates). Examples of these errors are shown in Supplementary Figure S10.

### 3.4 Increasing scale improves image classification accuracy

The accuracy of scale-dependent models for patch classification are presented in Figure 4C (extended results Supplementary Table S3).A peak AUC above 0.95 was observed when all training data were used. With training data from only 2–4 randomly selected slides, AUCs of 0.78–0.9 are observed. Average AUC increases rapidly from 0.88 for two slides to 0.94 for eight slides. Average AUC continues to increase from 8 to 49 slides but with much slower growth. Beyond 49 slides growth in average AUC continues but is modest. This asymptotic trend is often observed in machine learning experiments where orders of magnitude more data are needed to significantly improve performance near the asymptote.

## 4 Discussion

The success of convolutional networks in analyzing histology images has increased interest in strategies for producing annotation data. While ALs are demonstrating diagnostically meaningful performance in many applications, large amounts of annotations are required to develop and validate these models. This necessitates engaging multiple participants in annotation studies, and the ability to efficiently organize participants with a range of experience levels is one approach to scaling the annotation process. While significant expertise is needed for accurate semantic annotation of histology, our study provides an example application where non-experts can be trained to effectively perform much of the time-consuming work.

While non-experts cannot be expected to recognize rare patterns or to accurately annotate difficult cases, a large majority of the work in delineating tissue boundaries does not fall in these categories. By utilizing expertise where it is needed, in the annotation of rare or difficult classes, and in reviewing and correcting the annotations of non-experts, we were able to produce a large dataset containing over 20 000 annotated tissue regions. This resource can be used to train semantic segmentation models for breast cancer histology to characterize the tumor microenvironment and inflammatory infiltration, both of which are known to strongly correlate with cancer progression and patient outcomes (Fouad and Aanei, 2017).

The annotations in our study were produced using the DSA, a web-based digital pathology platform. While DSA provides a wide array of annotation tools, future development will enhance review and collaboration capabilities by formalizing these processes in specialized interfaces. These enhancements will increase the utility of DSA for annotation studies, education and diagnostic review including tumor boards.

Although concordance among participants was generally strong, important sources of discordance between SPs and NPs were observed (Supplementary Fig. S4). In the predominant classes, discordance was often observed in cases where judgment was either difficult or subjectively defined (e.g. a region is lymphocyte-rich if at least 80% of its area was occupied by lymphocytes). Less frequently occurring non-predominant classes were also often missed by NP participants, likely due to difficulty in recognizing these classes and a lack of training. Examples of annotation errors include stromal regions being mislabeled as necrosis or vice versa, hyalinized or acellular stroma misclassified as mucinous change, plasma cells being mislabeled as lymphocytes, and endothelial cells, activated fibroblasts or activated histiocytes being mislabeled as tumor. Missed classes include blood vessels, glandular secretions, as well as rare metaplastic changes and non-lymphocytic inflammatory infiltrates (it should be noted that much of the discordance arises from non-predominant classes added by the SPs during correction). We provide further evidence of the utility of NP annotations, showing that comparison models derived from NP annotations had similar accuracy to models derived from SP annotations. These comparison experiments were based on the limited set of ROIs for which both SP and NP annotations were available, and hence we still recommend supervision and feedback by SPs following the initial training of NP participants.

Semantic segmentation models derived from our annotation dataset were highly accurate, and provide new opportunities for feature extraction from breast cancer histology and tissue based studies. These models have a high macro-average AUC (0.897) and class-wise AUCs ranging from 0.881 (stroma) to 0.941 (inflammatory). Visualization shows that many of the areas where the human and computational prediction disagree is due to increased sensitivity of the models to granular regions that are not annotated by human participants.

While our study presents important findings on annotating histology images, there are a number of research questions that were not addressed. Our study relied on medical students and graduates, with the rationale being that basic familiarity with histology and general biology may reduce error rates. Future studies may investigate whether this assumption is correct, and if it is possible to engage a broader pool of participants that lack this training to further scale annotation efforts. Our study also did not evaluate intra-participant discordance, an issue that is known to be significant in pathology. Measuring intra-participant discordance would provide a baseline to evaluate inter-participant discordances against, and would provide better context for the differences in discordance observed among and between participants with different experience levels. The time participants spent in making annotations was also not recorded, nor was the time that more experienced users spent correcting annotations. This information, while difficult to acquire reliably, could provide further insights on how to best allocate resources in structured crowdsourcing studies. Finally, we would point out that the value of crowdsourcing likely varies by application. The amount of data required varies with the difficulty of the prediction task, whether the model is expected to generalize to specimens from different institutions, expectations for prediction model accuracy and the availability of experts to produce annotations. In some tasks, a well-resourced organization may be able to engage their pathologists to produce sufficient annotations for ‘in-house’ models not intended to generalize to specimens generated at other labs.

## Funding

This work was supported by the U.S. National Institutes of Health; and National Cancer Institute grants [U01CA220401, U24CA194362].

*Conflict of Interest*: Ventana did not fund this study, but they are funding others.

## Supplementary Material

btz083_Supplementary_Information
