# Supplementary material for: Structured crowdsourcing enables convolutional segmentation of histology images
Source: Bioinformatics. 2019 Feb 6;35(18):3461–7. doi: 10.1093/bioinformatics/btz083 (PMC6748796; doi:10.1093/bioinformatics/btz083)
Supplement: btz083_Supplementary_Information [file btz083_supplementary_information.zip › btz083-suppl_data/Supplementary_Information.12-29.MA.forUpload.pdf]

Structured Crowdsourcing Enables Convolutional Segmentation of Histology Images

Mohamed Amgad [1], Habiba Elfandy [2], Hagar H. Khallaf [3], Lamees A. Atteya [4], Mai A.T. Elsebaie [5], Lamia S. Abo Elnasr [6], Rokia A. Sakr [6], Hazem S.E. Salem [5], Ahmed F. Ismail [7], Anas M. Saad [5], Joumana Ahmed [3], Maha A.T. Elsebaie [5], Mustafijur Rahman [8], Inas A. Ruhban [9], Nada M. Elgazar [10], Yahya Alagha [3], Mohamed H. Osman [11], Ahmed M. Alhusseiny [10], Mariam M. Khalaf [12], Abo-Alela F. Younes [5], Ali Abdulkarim [3], Duaa M. Younes [5], Ahmed M. Gadallah [5], Ahmad M. Elkashash [3], Salma Y. Fala [13], Basma M. Zaki [13], Jonathan Beezley [14], Deepak R. Chittajallu [14], David Manthey [14], David A. Gutman [15], Lee A.D. Cooper [1,16]

[1]Department of Biomedical Informatics, Emory University School of Medicine, Atlanta, GA, USA, [2]Department of Pathology, National Cancer Institute, Cairo University, Cairo, Egypt, [3]Faculty of Medicine, Cairo university, Cairo, Egypt, [4]Egyptian Ministry of Health, Cairo, Egypt, [5]Faculty of Medicine, Ain Shams University, Cairo, Egypt, [6]Faculty of Medicine, Menoufia University, Menoufia, Egypt, [7]Medical Research Institute, Alexandria University, Alexandria, Egypt, [8]Chittagong University Faculty of Medicine, Chittagong, Bangladesh, [9] Faculty of Medicine, Damascus University, Damascus, Syria, [10]Faculty of Medicine, Mansoura University, Mansoura, Egypt, [11]Faculty of Medicine, Zagazig University, Zagazig, Egypt, [12]Faculty of Medicine, Batterjee Medical College, Jeddah, KSA, [13]Faculty of Medicine, Suez Canal University, Ismailia, Egypt, [14]Kitware Inc., Clifton Park, New York, USA, [15]Department of Neurology, Emory University School of Medicine, Atlanta, Georgia, USA, [16]Department of Biomedical Engineering, Emory University, Atlanta, GA, USA

Address correspondence to:

Lee AD Cooper, PhD
lee.cooper@emory.edu

SUPPLEMENTARY INFORMATION

SUPPLEMENTARY METHODS

Triple-Negative status assessment

Evidence of HER2/Neu status was obtained from IHC or FISH results in the clinical file, with positive HER2/Neu status being assigned to cases where there is disparity between the IHC and FISH studies.

Participant training process

Google suite tools, including Docs, Sheets, Slides, and Drive, were utilized to distribute training materials and slide assignments. A preliminary review of slides was first performed to describe their histologic subtypes and patterns. This information was captured in a spreadsheet to facilitate the assignment of slides to participants.

Annotation processing workflow

The DSA server stores polygonal annotations (including corrections) in a Mongo database in a coordinate list format. These coordinates are queried using the DSA REST API and are converted to a mask image format offline, where pixel values encode region class (Figure S1). This mask image conversion greatly simplifies the integration of corrections and calculation of inter-participant agreement statistics. Mask images are then processed to extract the updated polygonal coordinates that are pushed back to DSA for secondary review by the SPs and study coordinator.

Phases of review and correction

Two phases of review and corrections were used (Figure S3). During primary review, only the annotation polygon boundaries are displayed to maximize visibility of underlying tissue structures. This was meant to facilitate detection of major errors, such as polygon misclassifications or missing annotations, and is mainly done by the SPs. During secondary review, polygons are displayed in solid (filled) form, to give a better impression of what the final annotation masks will look like and to maximize visibility of minor artifacts and gaps. Minor corrections to polygon boundaries are made during this phase, mostly by the study coordinator.

Annotation discordance calculation and visualization

We summarize discordance using a statistic we describe as median slide-wise discordance:

$$\widetilde{\Delta} = median_{s1,..,10}(median_{ij1,...k}(\Delta_{i,j}))$$

Where Δ_(i,j,s) represents the discordance between participants i and j for evaluation slide s, and k represents number of participant pairs. This statistic first calculates the median discordance between participant pairs for each of the 10 evaluation ROIs, and then calculates the median across these discordances. The expected discordance between two participants on any given slide is represented by $\tilde{\Delta}$. We visualized inter-participant discordance directly on the images to determine where discordance between SPs and NPs occurs within evaluation set regions (Figure S4). To do this, we generated pixel-wise discordance maps using the following procedure for each region class: (1) SP masks were averaged (pixel-wise) to obtain soft ground truth masks. The averaged masks have values in the range [0,1], where 0.5 represents maximum discordance, and 0/1 represents maximum concordance. (2) Discordance masks were generated for each NP by taking the absolute difference between NP masks and the ground truth masks from step 1. (3) Perform pixel-wise averaging of the discordance masks from step 2 over all NPs to visualize the localization of SP-NP discordance in each region.

Fully-convolutional model training

The 16-layer, FCN-8 variant of VGG fully-convolutional network was used in our experiments. The network was trained to map pixels into five region classes: tumor, stroma, inflammatory infiltration, necrosis and other. Regions that belong to rare classes were grouped with predominant were classes where appropriate, as follows: Grouped with "tumor": angioinvasion, DCIS; Grouped with "inflammatory infiltrates": lymphocytes, plasma cells, other immune infiltrates. Each ROI was divided into overlapping 800x800 pixel tiles, and tiles where over 90% of the area was composed of the "don't care" class were ignored. The tiles were stored into *.tfrecords* files to be used in training. Slides from these institutes were not used in training the final segmentation model on the core set (i.e. were used as an unseen testing set to report accuracy): OL, LL, C8, BH, AR, A7 and A1. The amount of overlap used (and therefore, the amount of shift-augmentation) was inversely proportional to the size of the region of interest; this was done to ensure balanced representation of various histologic patterns regardless of ROI size. Crop augmentation to further increase robustness of training; a randomly-located 768x768 pixel image was cropped on the fly from each tile (after loading in memory) and was used in model training. The models were trained on 3 GPUs with a per-GPU batch size of 4 (total batch size of 12) using data parallelization. Adam optimizer was used with a

learning rate of 1e-5. Weighted categorical cross-entropy loss was used to mitigate class imbalance, with the weight associated with each class determined by:

$$W_c = \begin{cases} 0 & : if\ c = 0 \\ 1 - \frac{Nc}{N} & : if\ c > 0 \end{cases}$$

Where *N* is the total number of pixels in training dataset and *Nc* is total number of pixels belonging to class *c* in training dataset.

Patch classification model training

We trained a VGG-16 network to classify 224x224 pixel patches from the three predominant classes: tumor, stroma and inflammatory infiltration, using the same train/test assignment used in the semantic segmentation model. Each ROI was divided into non-overlapping patches at 20x objective magnification, and patches where the majority class falls below the 50% area were discarded as ambiguous. The convolutional layers and first fully-connected layer were derived from the pre-trained ImageNet VGG-16 network and fixed as non-trainable. Two fully-connected layers were added to the fixed network and trained using cross-entropy loss in TensorFlow. A static testing set was derived from 43 ROIs (13,888 patches), while a variable number of the remaining ROIs were randomly selected for training.

Statistical tests

Mann-Whitney U test was used for unpaired comparisons, and Wilcoxon signed-rank test was used for paired comparisons. A threshold level of 0.05 was used to determine statistical significance.

SUPPLEMENTARY TABLES

Table S1: Guiding instructions given and tips learned from our experience.

Instruction	Rationale
**General instructions**	
Explain importance of annotation quality for algorithm training. Emphasize importance of accurate conformation to region boundaries.	Some participants may have a misconception that their annotations are only meant to indicate "where the tumor generally is", rather than accurately delineating boundaries.
Explain importance of comfortable workstation, including correct posture, frequent breaks, and mouse usage.	The health and comfort of the participants is important in its own right, and is critical to ensure compliance and high quality annotations.
Provide rules about general diagnostic workflow - zoom in and out and pan the slide for general orientation. Provide criteria on minimum and maximum magnification for annotating classes.	Some region classes are easier to recognize at lower magnification, such as infiltrating lymphocytes which have a "salt and pepper" appearance. Very low magnifications can result in inaccurate boundaries, while very high magnifications significantly increase workload, potentially degrading quality or reducing compliance..
Provide an unambiguous template of histological classes to annotate, including definitions of those classes and how they can be effectively recognized.	This helps standardize the annotation process, reduces variability, and guards against common confusions made by novices.
**Instructions to prevent annotation mask artifacts**	
When a region extends beyond the ROI, extend the annotation to slightly overlap the ROI boundary.	This prevents gaps between region polygons and the ROI boundary in the annotation mask (See **Figure 3B** and **Supplementary Figure 1**).
When a region is too large to enclose in a single polygon, use multiple overlapping polygons.	This avoids inaccuracies caused by participant fatigue. Overlapping annotations of the same region class are fused offline.
Minimize gaps between annotations of different region classes.	This increases accuracy of resultant masks and avoids misclassification of pixels at the interface between non-background region classes as background.
When two regions are enclosed within one another (eg lymphocytic infiltrate within a tumor region), make sure the polygon boundaries are also completely enclosed and non-crossing.	This facilitates conversion of polygonal coordinates into masks by detecting the hierarchy of polygon enclosure (and hence, overlay order) using common image analysis libraries (**Figure 3B**).
Clear definition of "baseline" or background class	Having a default background class (in our case, stroma) significantly reduces the annotation workload and reduces chances of error.
Clear rules about what constitutes a legal polygon: 1- Moderate-sized closed polygons preferred, even if this means having multiple overlapping polygons to enclose a single anatomical structure; 2- Keep mental image of where the interior of a polygon is; 3- Avoid self-crossing polygon boundaries.	Illegal or malformed polygons are difficult to handle and correct offline, and may significantly degrade the quality of the resultant masks.

Table S2: Number of annotations in final dataset, broken down by region class. * stromal polygon counts only reflect stroma enclosed within non-stromal regions. Other stromal areas are considered the default background class so no polygons were extracted for them.

Broad category	Region class	Annotation count (%)
Predominant classes	Tumor	6536 (32.1%)
	Stroma *	2531 (12.4%)
	Lymphocyte-rich	5066 (24.9%)
	Necrosis or debris	506 (2.5%)
Non-predominant classes	Exclude (artifacts, tears, empty lumina, etc)	1943 (9.6%)
	Adipose tissue (fat)	1108 (5.4%)
	Blood vessel	633 (3.1%)
	Blood (intravascular or extravasated red blood cells)	611 (3.0%)
	Glandular secretions	93 (0.5%)
	Extracellular mucoid material	63 (0.3%)
Challenging classes	Plasma cells	806 (4.0%)
	Mixed inflammatory infiltrates	122 (0.6%)
	Metaplastic changes (osteoid,cartilaginous matrix etc)	4 (0.0%)
	Lymph vessel	11 (0.1%)
	Skin adnexa	2 (0.0%)
	Angioinvasion	12 (0.1%)
	Nerves	1 (0.0%)
	DCIS	9 (0.0%)
	Normal acinus or duct	138 (0.7%)
	Undetermined (eg cannot be determined without IHC)	145 (0.7%)

Table S3: Patch classification AUC and accuracy improves with larger training datasets. Summary of area under receiver-operator characteristics curve (ROC AUC) and accuracy of a convolutional neural network trained to classify patches into tumor, stroma, and inflammatory classes. Each row represents a set of 10-20 experiments where a fixed number of randomly chosen ROIs/slides were assigned to the training set. Number of patches is variable because the size of the ROIs varies between different slides. Numbers presented represent the mean and standard deviation (in brackets) of classification accuracy and AUC on a static testing set composed of 43 ROIs (13,888 patches).

No. of training ROIs	No. of training patches	AUC (Macro-average)	AUC (tumor)	AUC (stroma)	AUC (inflammatory)	Accuracy (overall)	Accuracy (tumor)	Accuracy (stroma)	Accuracy (inflammatory)
2	778.40 (389.94)	0.88 (0.04)	0.88 (0.04)	0.88 (0.03)	0.89 (0.08)	0.73 (0.04)	0.79 (0.04)	0.79 (0.03)	0.88 (0.04)
4	1526.10 (468.80)	0.92 (0.01)	0.92 (0.02)	0.90 (0.02)	0.94 (0.01)	0.78 (0.03)	0.83 (0.03)	0.82 (0.02)	0.90 (0.03)
5	1661.20 (437.09)	0.92 (0.01)	0.92 (0.02)	0.92 (0.01)	0.94 (0.01)	0.78 (0.03)	0.84 (0.03)	0.84 (0.01)	0.90 (0.02)
8	2691.00 (312.69)	0.94 (0.01)	0.94 (0.01)	0.92 (0.01)	0.95 (0.01)	0.81 (0.01)	0.86 (0.01)	0.84 (0.01)	0.92 (0.01)
9	2982.90 (498.45)	0.93 (0.01)	0.93 (0.01)	0.92 (0.01)	0.94 (0.01)	0.80 (0.01)	0.84 (0.01)	0.85 (0.01)	0.92 (0.00)
12	4496.70 (1338.68)	0.94 (0.01)	0.94 (0.01)	0.92 (0.02)	0.95 (0.00)	0.80 (0.05)	0.85 (0.03)	0.85 (0.02)	0.90 (0.04)
16	5160.60 (663.00)	0.94 (0.00)	0.94 (0.01)	0.93 (0.01)	0.95 (0.01)	0.81 (0.01)	0.86 (0.01)	0.84 (0.02)	0.92 (0.01)
32	10854.00 (1016.51)	0.95 (0.01)	0.95 (0.00)	0.93 (0.02)	0.96 (0.00)	0.81 (0.03)	0.87 (0.01)	0.85 (0.02)	0.90 (0.04)
41	14493.10 (1116.62)	0.95 (0.00)	0.95 (0.00)	0.94 (0.00)	0.96 (0.00)	0.83 (0.01)	0.87 (0.01)	0.86 (0.01)	0.92 (0.00)
49	17091.40 (755.45)	0.95 (0.00)	0.95 (0.00)	0.94 (0.00)	0.96 (0.00)	0.83 (0.02)	0.88 (0.01)	0.86 (0.01)	0.92 (0.01)
65	23284.10 (465.63)	0.95 (0.00)	0.96 (0.00)	0.94 (0.00)	0.96 (0.00)	0.82 (0.02)	0.87 (0.02)	0.85 (0.03)	0.93 (0.00)
82	28541.00 (0.00)	0.95 (0.00)	0.96 (0.00)	0.94 (0.00)	0.96 (0.00)	0.83 (0.01)	0.88 (0.01)	0.86 (0.02)	0.92 (0.00)

SUPPLEMENTARY FIGURES

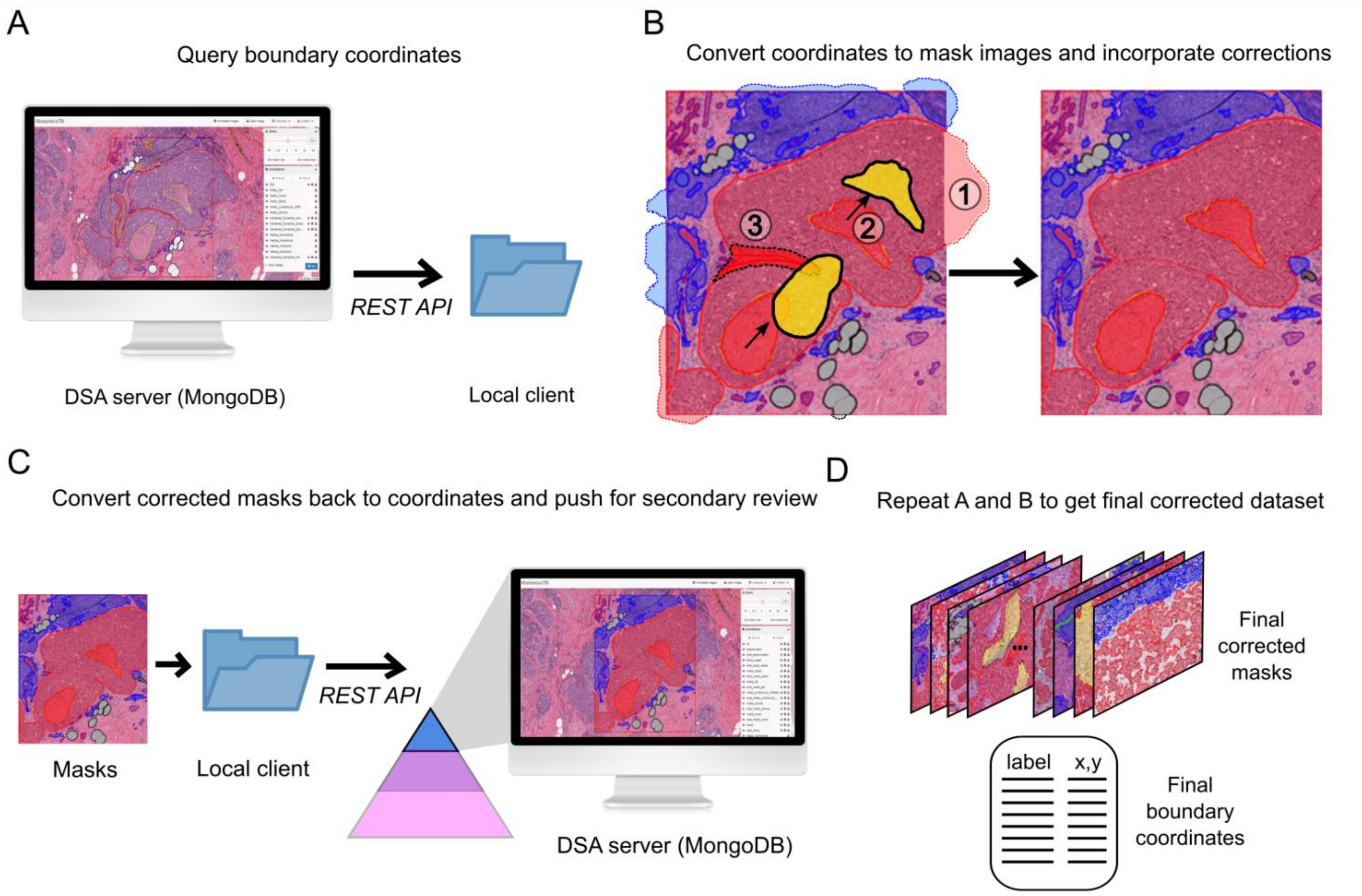

Figure S1: Processing annotations and integrating corrections. (A) The DSA server stores annotations and corrections as polygonal coordinates. The process of integrating corrections and generating masks begins by downloading these polygonal descriptions from the server using the REST API provided by DSA. Making corrections and calculating inter-participant agreement is more easily performed with mask images than with raw polygonal coordinates, while polygons are easier to store in the DSA database. **(B)** Before integrating corrections, annotations are converted to mask label images in a process that includes 1. Cropping polygons to the ROI boundary 2. Determining order of enclosure (polygons enclosed within other polygons are overlaid on top) and 3. Fusing the correction mask image with uncorrected mask image. **(C)** The fused mask images are then converted to polygons and uploaded back to DSA using the REST API. **(D)** Steps A and B are repeated to obtain final corrected dataset.

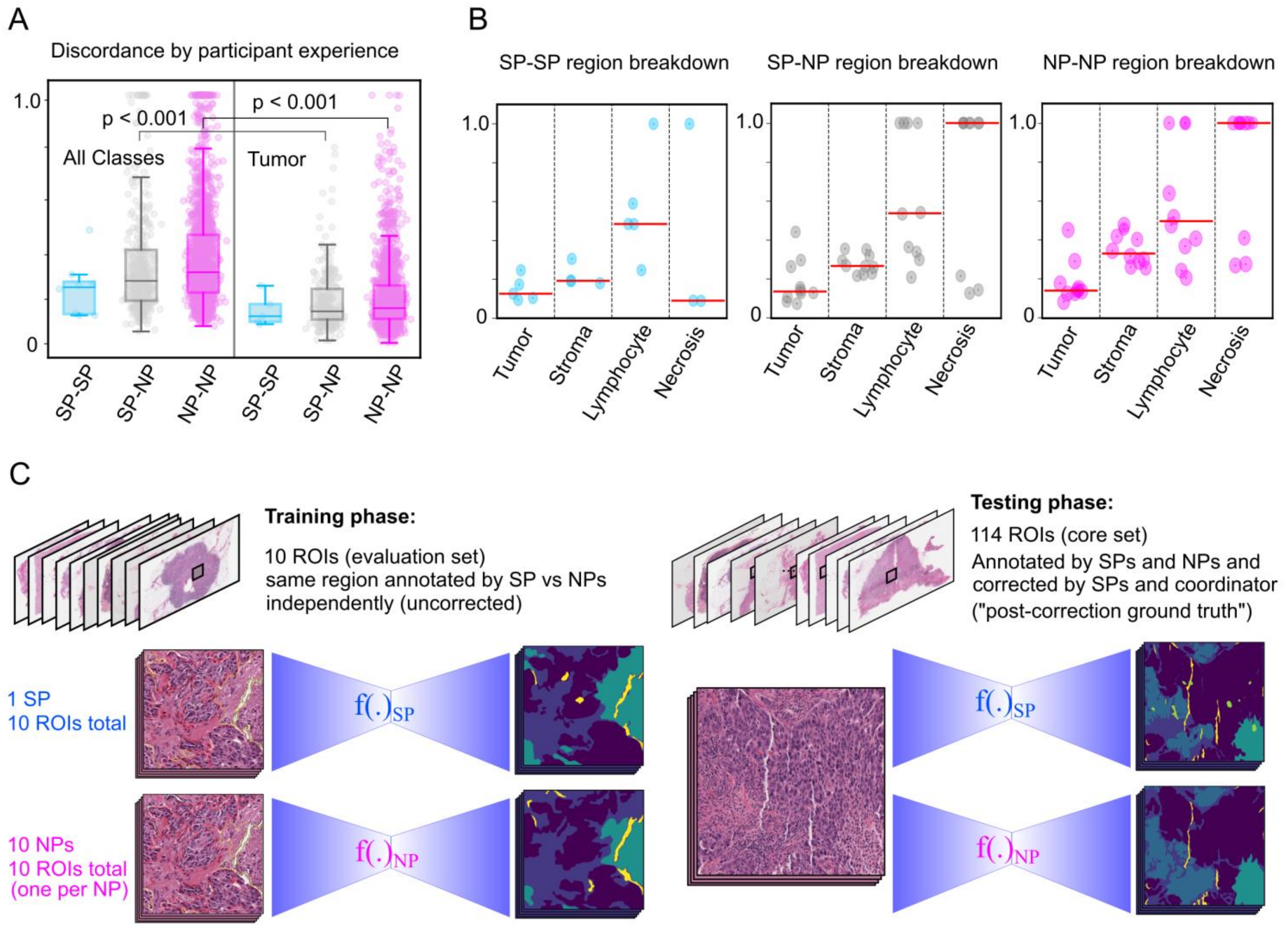

Figure S2: Evaluation slide set discordance and model training process. (A) Discordance by participant experience. Each point represents the discordance between one participant pair for a single slide. **(B)** Slidewise discordance breakdown by region class. The position of each circle represents the median inter-participant discordance for one slide and one region class. The diameter of the circle is proportional to the number of masks aggregated, eg. some necrotic regions are annotated by some participant pairs, but not others. The red lines indicate the $\tilde{\Delta}$ value. **(C)** Investigating the impact of variability and lack of experience on model accuracy. **Left** - Two training sets were used, one based on a single SP evaluation set annotations, and another based on 10 NP annotations using the same evaluation set ROIs. While some vetting was done by the study coordinator (choosing which mask from which NPs to include), these annotations were used as is. **Right** – The two trained models, one based on a single SP and another based on 10 NPs were evaluated against 114 infiltrating ductal post-correction ground truth annotations from the core slide set.

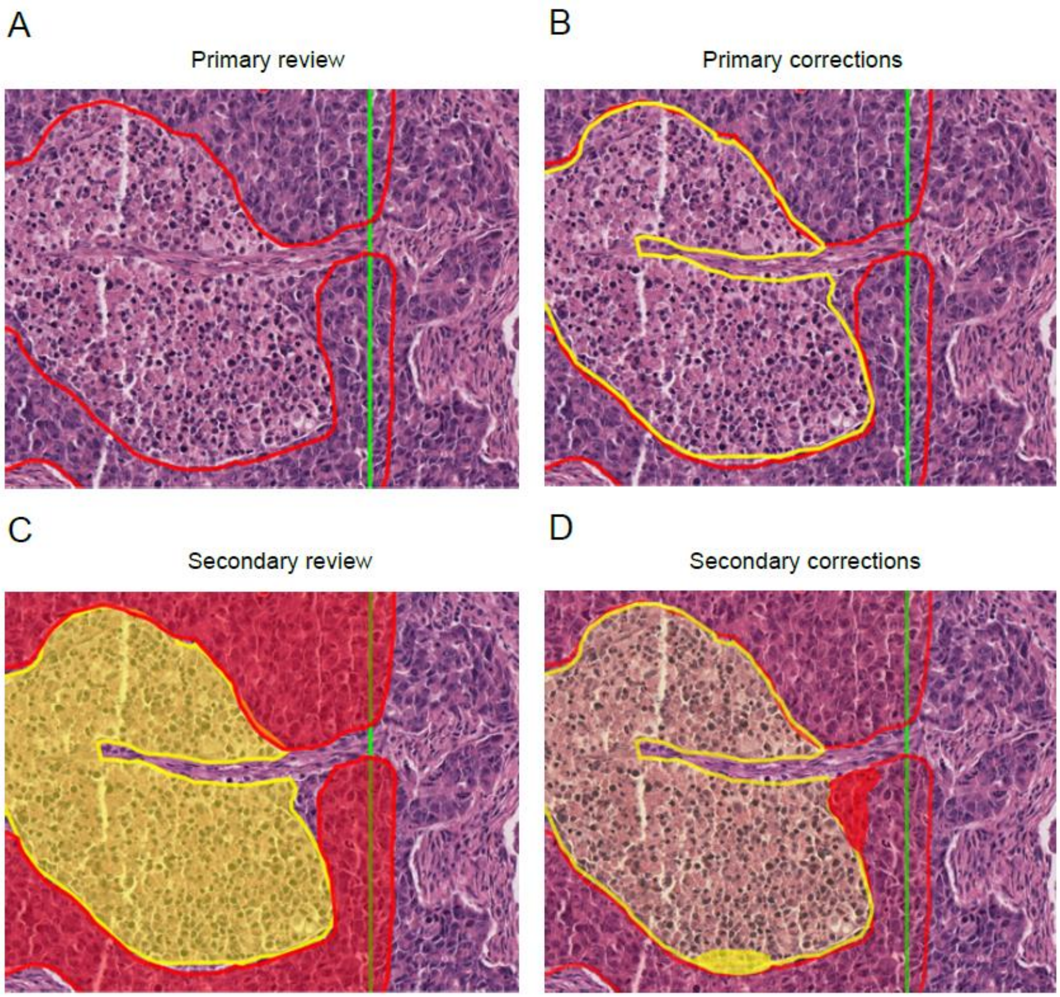

Figure S3: Two-stage review and correction process. The vertical green line represents the region-of-interest boundary. Notice how the participant extended his/her tumor annotation slightly beyond the green region-of-interest boundary in accordance with the annotation instructions in Supplementary Table 1. **(A)** Primary review process involves visualizing the annotation polygon boundaries without fill. **(B)** Major corrections, in this case a missing necrosis/debris region, are made during primary review. **(C)** Secondary review process involves visualization of solid polygons after incorporation of primary corrections. **(D)** Any gaps or artifacts are corrected during secondary review.

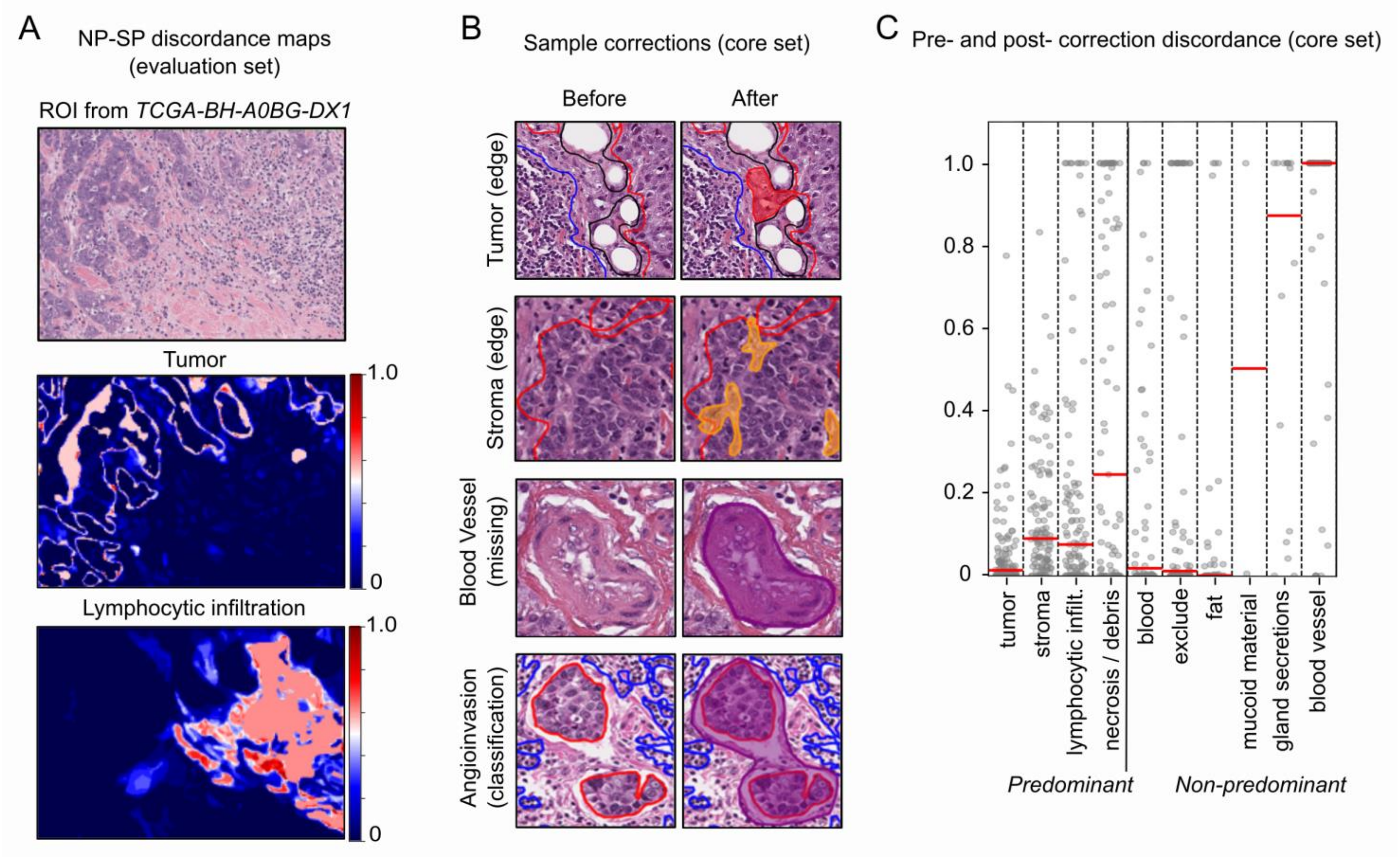

Figure S4: Sources of errors and the annotation correction process. (A) Pixel-wise discordance between senior pathologists and non-pathologists on two evaluation set slides (see supplementary methods). **(B)** Sample corrections on the core slide set. Original annotations appear as polygon boundaries, while corrections appear as solid polygons. **(C)** Discordance between pre-correction and post-correction masks for non-pathologists in the core set. The position of each dot represents the median inter-participant discordance for one slide and one region class. The red lines indicate the overall $\tilde{\Delta}$ value per region class.

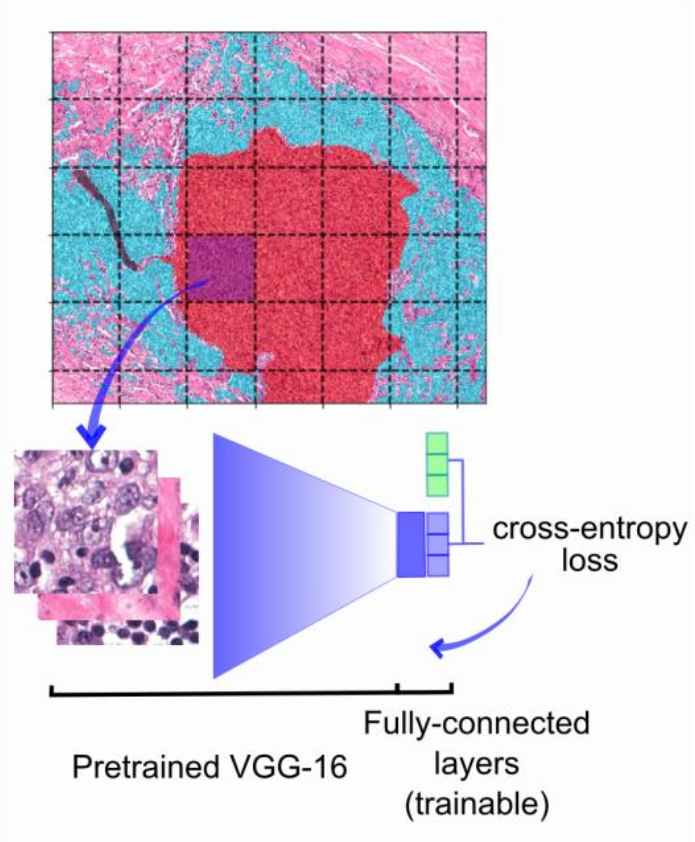

Figure S5: Investigating effect of training dataset size on model generalization. We trained a convolutional neural network to classify patches by pixel class majority into three predominant classes (tumor, stroma, and inflammatory infiltrate). An Imagenet-pretrained VGG-16 architecture was used, with non-trainable weights up to and including the first fully-connected layer, and two trainable fully-connected layers.

.

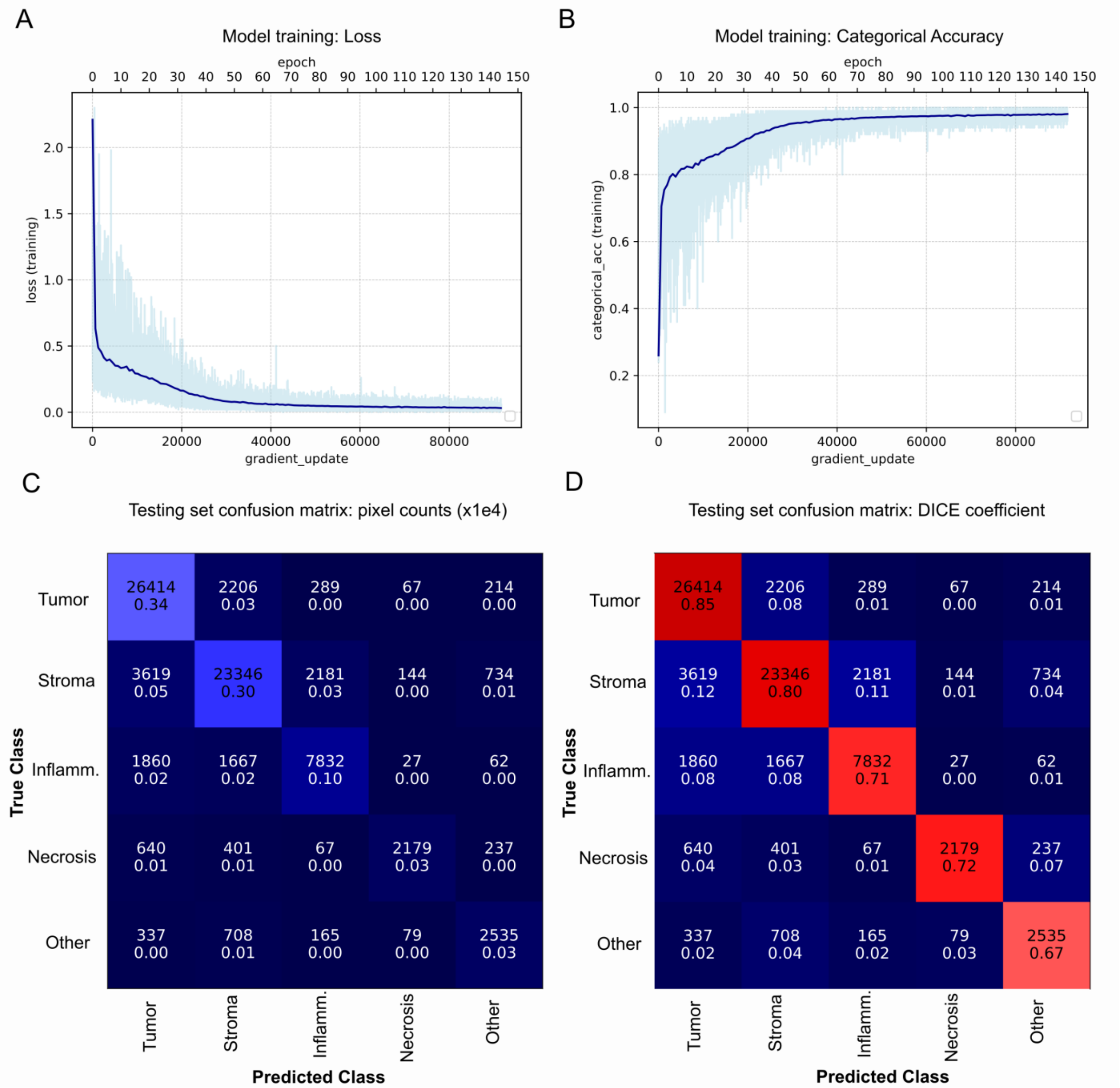

Figure S6: Semantic segmentation accuracy. (A) Loss over the training set. **(B)** Categorical accuracy over the training set. **(C)** Confusion matrix over testing set, normalized to pixel counts. Top numbers are pixel counts (x1e4) and bottom numbers represent fraction of total pixel counts. **(B)** Confusion matrix over testing set using DICE statistic. Top numbers are pixel counts (x1e4) and bottom numbers represent DICE coefficient.

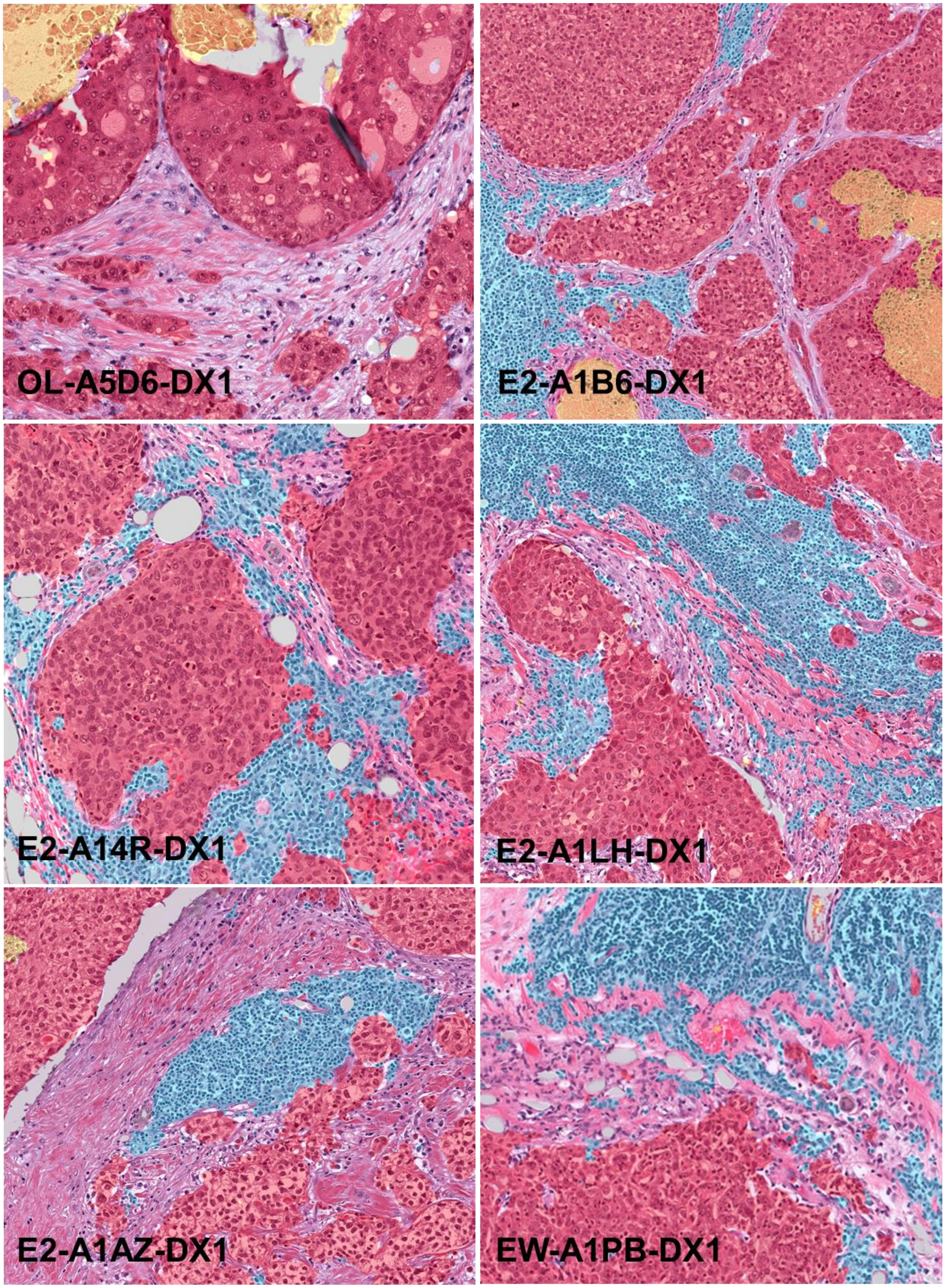

Figure S7: Semantic segmentation visualization over selected testing set ROI's (1). Color codes used: red (tumor); transparent (stroma); cyan (inflammatory infiltrates); yellow (necrosis).

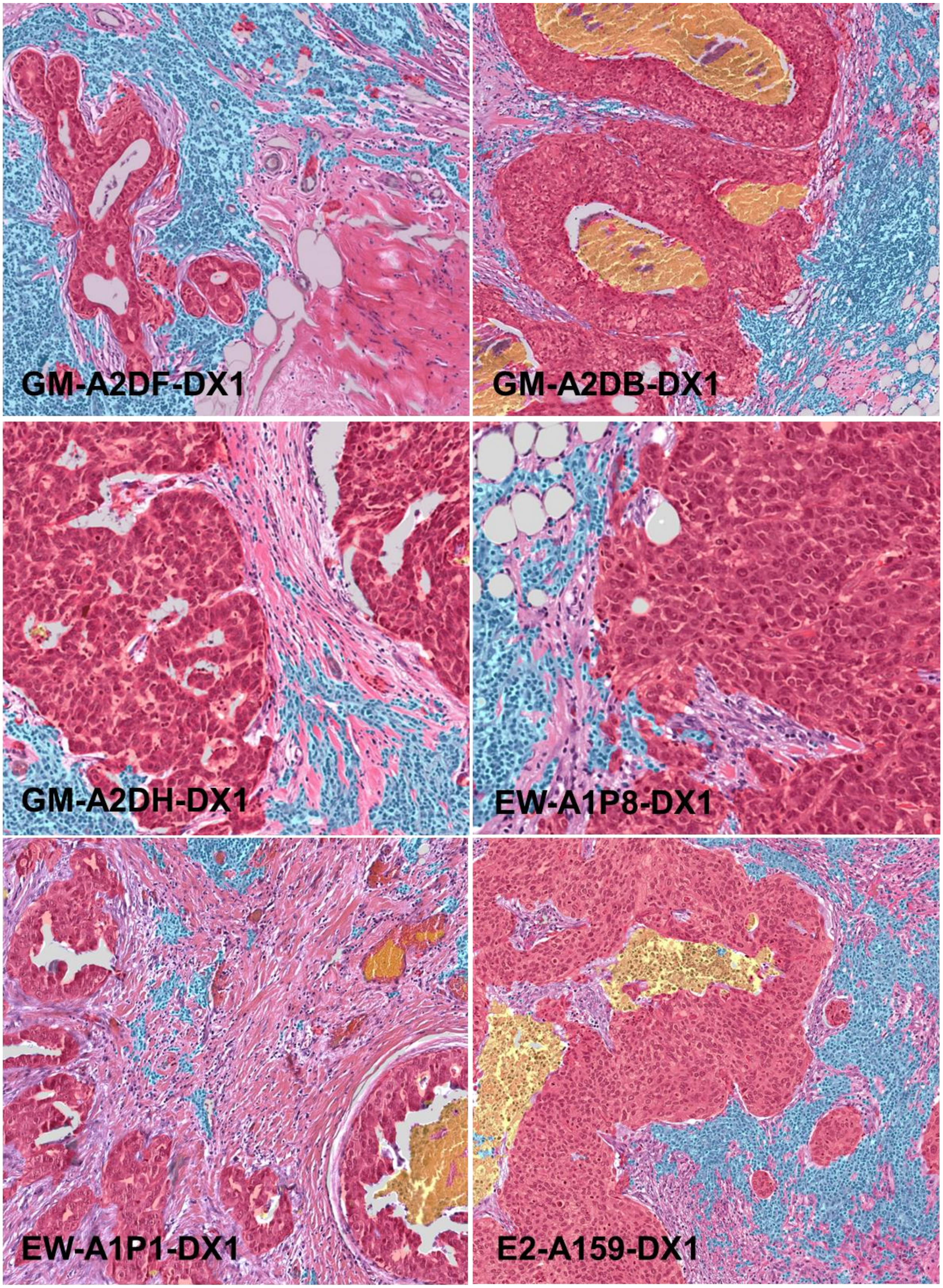

Figure S8: Semantic segmentation visualization over selected testing set ROI's (2). Color codes used: red (tumor); transparent (stroma); cyan (inflammatory infiltrates); yellow (necrosis).

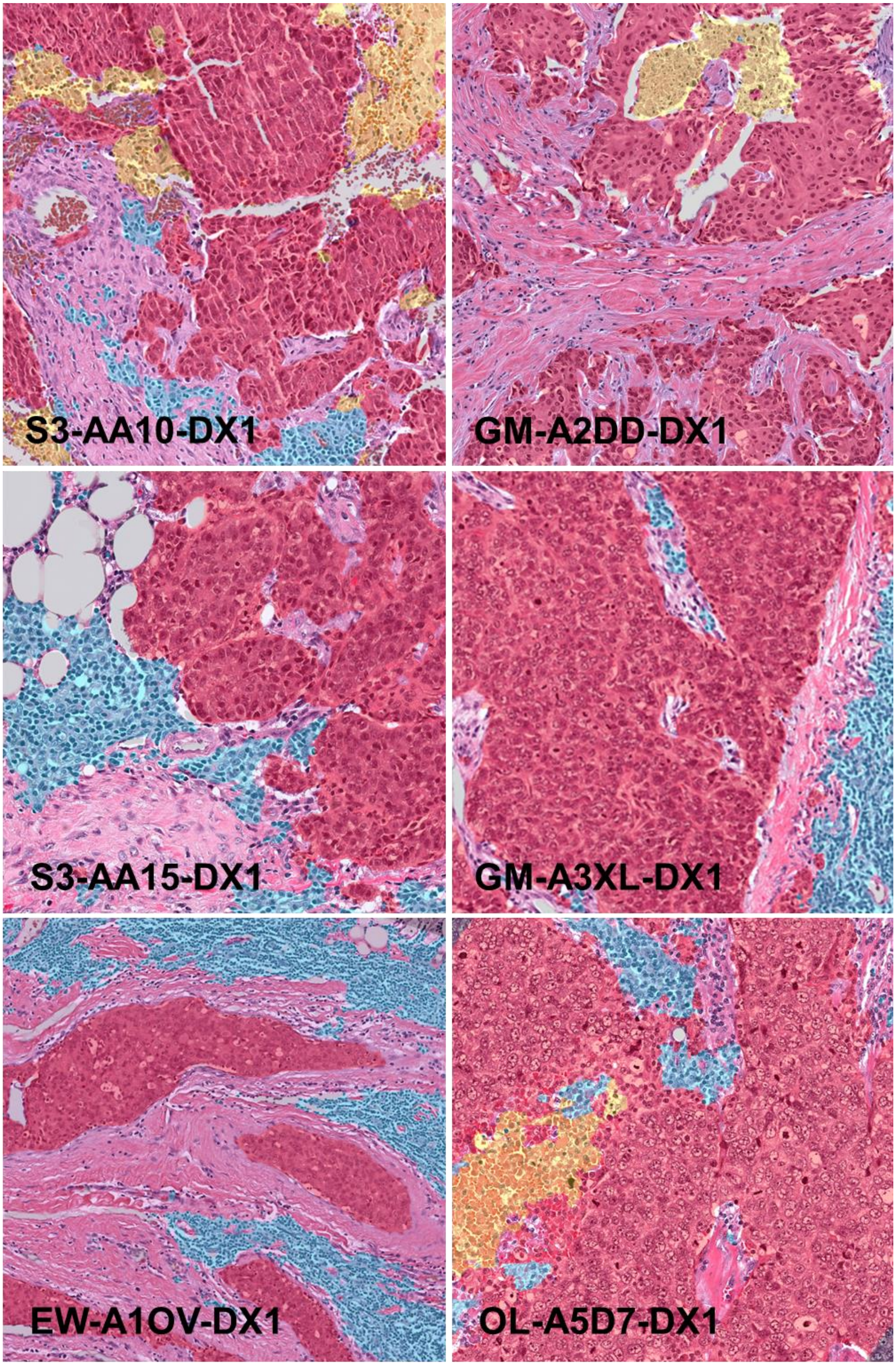

Figure S9: Semantic segmentation visualization over selected testing set ROI's (3). Color codes used: red (tumor); transparent (stroma); cyan (inflammatory infiltrates); yellow (necrosis).

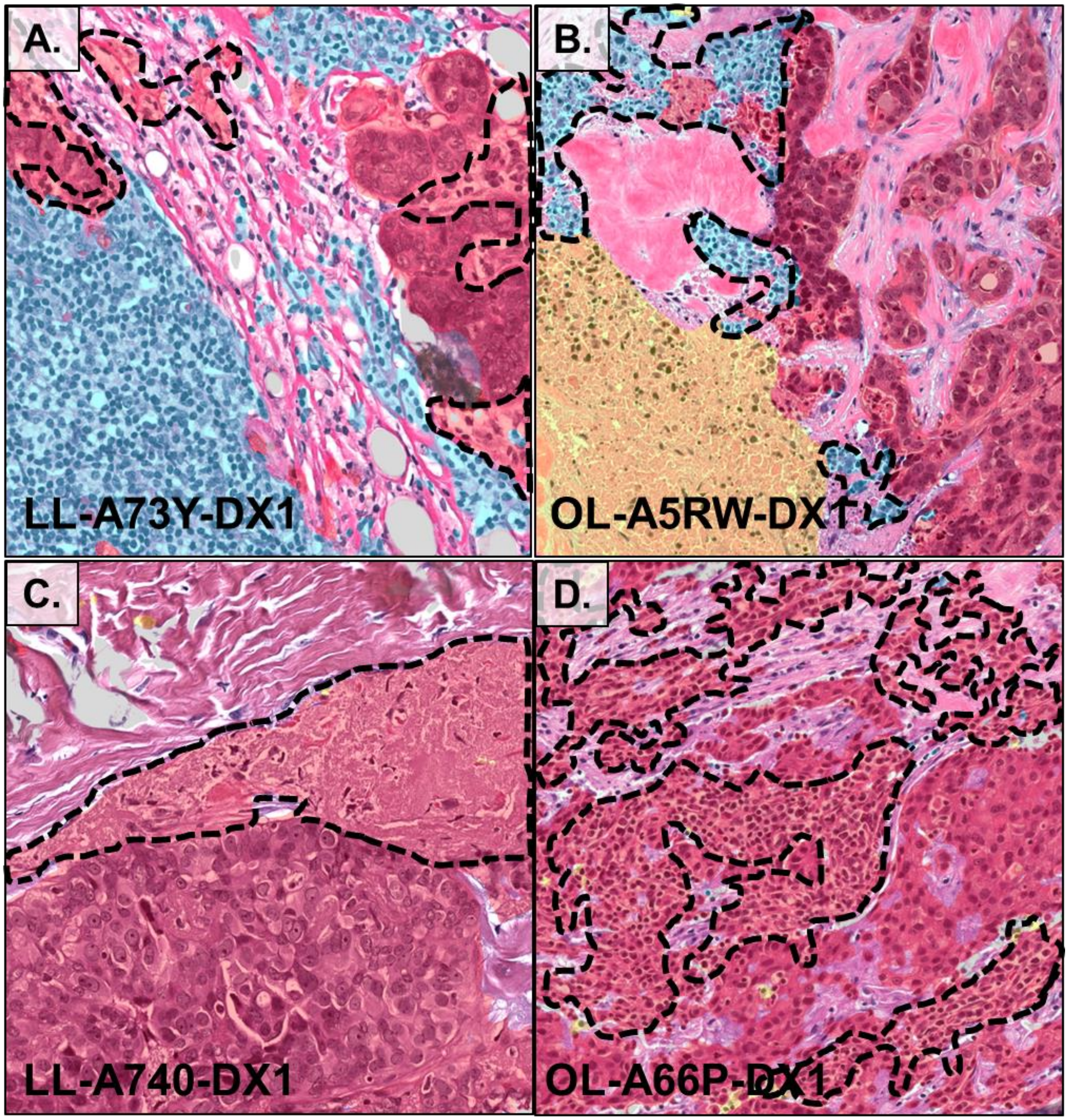

Figure S10: Patterns where semantic segmentation algorithm departs from ground truth annotations (testing set). (A) Stroma intervening small tumor nests misclassified as tumor. **(B)** Necrotic regions infiltrated by inflammatory cells classified as inflammatory infiltrates. The dataset was constructed such that these patterns are identified as necrosis instead. From a pathology standpoint, this is not a misclassification, though it does differ from ground truth and contributes to accuracy metrics. **(C)** Hyalinized, acellular stroma misclassified as tumor. This pattern is uncommon and was not represented during model training. **(D)** Dense plasma cell infiltrates misclassified as tumor. Plasma cells were most commonly present in admixtures with other inflammatory cells, especially lymphocytes, during model training. This pattern is also uncommon and was not represented during model training.

SUPPLEMENTARY DATASET

Dataset visualization (*Kitware*): The curated dataset can be visualized at the following public instance of the Digital Slide Archive from *Kitware Inc.*:

http://demo.kitware.com/histomicstk/histomicstk#?image=5bbdee62e629140048d01b0d

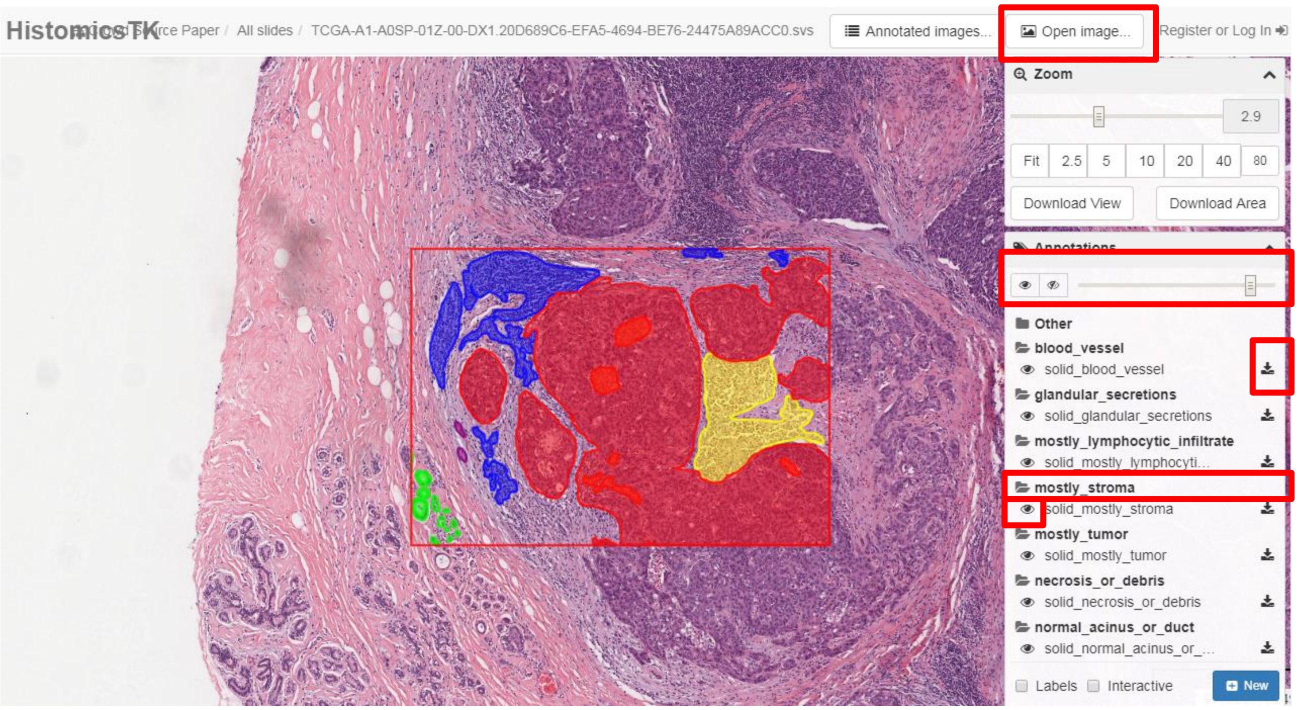

To view a specific slide, click *"Open image"* in the upper right corner and choose the *"Crowdsource paper"* collection. Use the *eye icon* under the "Annotations" tab to view all annotations, and use the *slide bar* to control annotation transparency. To view any particular group of annotations, click on the corresponding *folder icon*, then click the eye icon to toggle visibility. You may download any individual annotation by clicking the *downward arrow* symbol, although it is probably easier to access the ground truth masks directly using the link and instructions below. *Note: for visualization purposes, stromal regions were only explicitly annotated if enclosed within another region class.*

Ground truth masks: The ground truth masks for this dataset (to be used for model training and validation) can be found at the following link: *https://figshare.com/s/eae85d914fcb2920da23*. Each mask is a *.png* image, where pixel values encode region class membership. The meaning of ground truth encoded can be found at the file *gtruth_codes.tsv* found in the same directory. The name of each mask encodes all necessary information to extract the corresponding RGB images from TCGA slides, as follows:

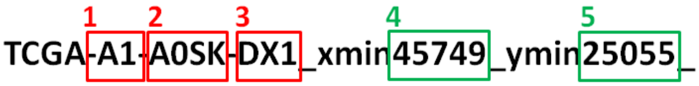

***1.** Center/hospital where the patient was treated; **2.** TCGA unique patient I.D.; **3.** Slide I.D.; **4.** Minimum x-coordinates in pixels relative to slide .svs file (at native magnification); **5.** Minimum y-coordinates in pixels relative to slide .svs file (at native magnification).*

Please be aware that some of the regions of interest are rotated, and that zero pixels represent regions outside the region of interest ("don't care" class) and should be assigned zero-weight during model training; they do not represent an "other" class. This rotation was done in the interest of capturing adjacent, yet diverse histologic patterns with minimal annotator fatigue.

Supplementary_Tables.xlsx: Raw data and tables used for concordance analysis and convolutional network accuracy reporting. This excel file contains various sheets, described below:

- **Concordance_evaluation_set:** Concordance statistics for participant pairs over the evaluation set. The columns have the following meaning:
  - *Slide_name*: name of the slide from which the evaluation ROI was taken.

  - *Participant1* / *Participant2*: identifier for study participant who performed the annotation.
  - *Label*: Region class for which the concordance was calculated.
  - *Intersect*: Intersection of the two binary masks. This is the number of pixels commonly classified by both participants as belonging to the label of interest.
  - *Sums*: Bag union of the two binary masks. This is the sum of annotated pixels by each of the participant pairs.
  - *Dice*: Dice coefficient.

- **Concordance_core_set:** Concordance statistics of pre- and post- correction masks. The columns have the same meaning as the evaluation set concordance set.

- **Patch_CNN_testing_accuracy:** CNN patch classification testing accuracy and AUC for each experiment. The columns have the following meaning:
  - *N_slides_train:* Number of ROI's (each from a unique slide) in the training set.
  - *N_patches_train:* Number of patches in the training set.
  - *Accuracy:* Overall accuracy.
  - *Accuracy_tumor / Accuracy_stroma / Accuracy_inflammatory:* Accuracy breakdown by patch class.
  - *ROCAUC:* Macro-averaged area under receiver operator characteristics curve over testing set.
  - *ROCAUC_tumor / ROCAUC_stroma / ROCAUC_inflammatory:* ROCAUC breakdown by patch class.

- **FCN_AUC:** Area under ROC curve for softmax pixel values for each slide in the testing set, broken down by region class.

- **FCN_confusion:** Overall confusion matrix over the testing set, numbers represent pixel counts.
